# Transition from ripples to faceted structures under low-energy argon ion bombardment of silicon: understanding the role of shadowing and sputtering

**DOI:** 10.1186/1556-276X-8-289

**Published:** 2013-06-19

**Authors:** Tanmoy Basu, Debi Prasad Datta, Tapobrata Som

**Affiliations:** 1 SUNAG Laboratory, Institute of Physics, Sachivalaya Marg, Bhubaneswar 751 005, India

**Keywords:** Silicon, Ion beam patterning, Atomic force microscopy

## Abstract

**Abstract:**

In this study, we have investigated temporal evolution of silicon surface topography under 500-eV argon ion bombardment for two angles of incidence, namely 70° and 72.5°. For both angles, parallel-mode ripples are observed at low fluences (up to 2 × 10^17^ ions cm^-2^) which undergo a transition to faceted structures at a higher fluence of 5 × 10^17^ ions cm^-2^. Facet coarsening takes place at further higher fluences. This transition from ripples to faceted structures is attributed to the shadowing effect due to a height difference between peaks and valleys of the ripples. The observed facet coarsening is attributed to a mechanism based on reflection of primary ions from the facets. In addition, the role of sputtering is investigated (for both the angles) by computing the fractional change in sputtering yield and the evolution of surface roughness.

**PACS:**

81.05.Cy, 81.16.Rf, 61.80.Jh, 87.64.Dz

## Background

Low-energy ion beam sputtering (IBS) is considered to be a very promising and cost-effective technique to fabricate self-organized nanoscale periodic patterns on a large-area (up to 2- to 3-in. diameter) solid surface in a single step [1]. Such nanoscale periodic structures (mostly ripples) are considered to be useful as templates for growth of nanofunctional thin films having potential applications in plasmonics, nanoscale magnetism, and other technological applications. For instance, Ag films deposited on rippled silicon substrate show strong optical anisotropy [2,3] and Fe films on rippled substrates demonstrate magnetic anisotropy which are driven by morphological anisotropy [4,5]. Direct nanoscale ripple patterning can also induce in-plane uniaxial magnetic anisotropy in epitaxial [6] and polycrystalline ferromagnetic Fe or Ni films [7]. In another study, it has been shown that rippled Au films show anisotropy in electrical transport property [8].

It is well established that ripple characteristics depend on beam and target parameters, namely ion species, ion energy, ion flux, ion fluence, ion incident angle, composition, and sample temperature [9-17]. In addition, experimental studies have shown that evolution of ion beam-induced ripple morphology is related to continuous change in sputtering yield even at any given angle [18-20]. For instance, Stevie et al. reported that in the case of ripple formation at 52° (for 6 keV O^2+^ ions), the sputtering yield got enhanced by nearly 70% as compared to the initial value [21]. However, an accurate prediction of change in sputtering yield is still not well developed due to a complex nature of the problem (i.e. complex mechanisms leading to a surface morphology and the existing interplay between these mechanisms and change in sputtering yield).

In addition to the experimental studies, there exist substantial amount of theoretical studies to explain IBS-induced ripple formation. Bradley-Harper (B-H) theory and its extensions were invoked to explain ion erosion-induced ripple formation due to off-normal ion bombardment and its coarsening [22,23]. Following these theories, there are reports which show that although ripples are more or less periodic in nature in the linear regime, with increasing time, it may change to a sawtooth-like morphology [9,12,13]. This type of transition from ripples to sawtooth or faceted structures was mentioned by Makeev and Barabasi for small surface gradients [24,25] which was later generalized by Carter at intermediate ion energies (few tens of kiloelectron volts) for all surface gradients [26]. In his work, Carter showed (based on geometrical argument) that with the growth of ripple amplitude-to-wavelength ratio, the shadowing of the incident ion beam by surface features can lead to formation of sawtooth-like facets. In a later work, this model was applied to explain morphological transition observed under bombardment of silicon by 30 keV argon ions [27]. However, applicability of this very approach is yet to be explored for low-energy (hundreds of electron volts) ion-induced transition from ripples to faceted structures under continuous ion bombardment. A major reason for this is the lack of available experimental data on the formation of faceted structures using low-energy ions.

For instance, Keller and Facsko reviewed the temporal evolution of ripple formation on Si by low-energy Ar ion bombardment [28]. They compared the predictions of various continuum models with experimentally observed ripple morphologies. In a previous work, Ziberi et al. reported well-ordered ripple formation on Si surface by 1,200 eV argon ion bombardment at 15° [29]. This contradicts the results of Keller and Facsko where the surface remained stable at near-normal incidence of Ar ions. In another work, Frost et al. reported on various pattern formations (ripples, dots, and their combination) and smoothening of silicon surface by low-energy ion beam erosion [30]. The effect of elevated target temperature during ion beam sputtering was addressed by Brown et al. [31]. Evolution of surface morphology during 500 eV Ar ion-induced erosion of Si(111) at an oblique incidence of 60° was demonstrated over a temperature range of 773 to 1,003 K. Formation of dots with rectangular symmetry was reported at temperatures above 963 K, whereas perpendicular-mode ripples were observed below this temperature. Thus, there is a room to look for controlled synthesis of self-organized faceted structures on silicon surface using similar ion energies.

In this study, we report on the transition from ripples to faceted structures on silicon surface beyond a threshold ion fluence and their coarsening at even higher fluences. As a novelty, we study this transition in the unexplored low ion energy regime which is roughly two orders of magnitude lower than those studied in the aforementioned works [9,12,13,26,27]. In this energy regime, smaller ion penetration depth, ion-mediated amorphization, and sputtering yields may lead to different pattern formation and dynamics. We have selected two different oblique incident angles, namely 70° and 72.5°.

In addressing the mechanism of the observed transition, variation in the erosion rate of a sinusoidal surface is calculated using the theoretical model of Carter [26]. It is seen that for critical values of the amplitude-to-wavelength ratio, inter-peak shadowing of incident ion flux can lead to a transition from ripples to faceted structures. The coarsening behaviour of faceted structures with increasing fluence is explained in light of Hauffe’s mechanism based on reflection of primary ions [32]. Carter’s theory is also used to calculate fractional change in sputtering yield for ripples and faceted structures (where we have replaced the amplitude-to-wavelength ratio by amplitude-to-base width ratio for the latter ones) which is observed to follow a nearly similar trend as that of surface roughness as the evolution of the observed patterns takes place.

### Theoretical approach

**Figure 1:**
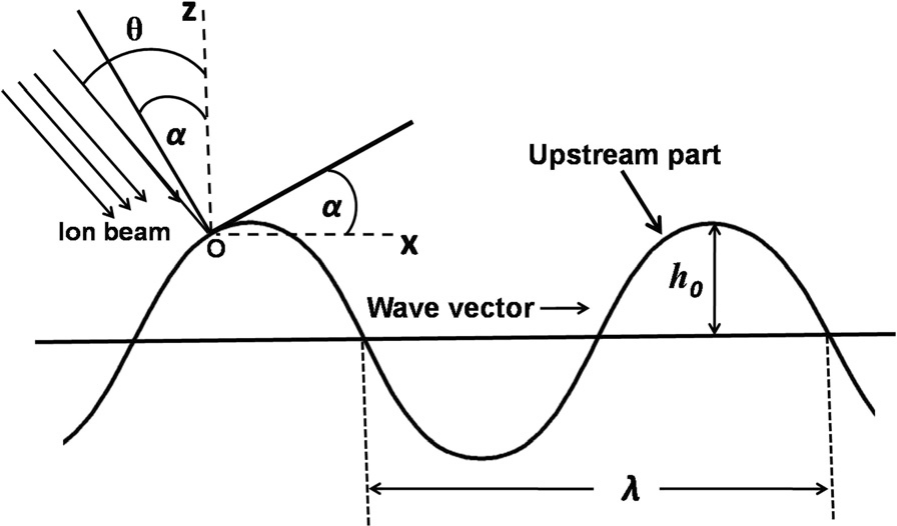
**Ion bombardment of a sinusoidal wave geometry.** Ion flux density, *J*, incident at an angle *θ* with respect to mean surface plane is shown. Local surface gradient, tan $\alpha =\left(\frac{\partial h}{\partial x}\right)$. Sinusoidal wave is described by *h* = *h*_0_ sin(2*πx/λ*), where *λ* is the wavelength of the ripples, and *h*_0_ is the amplitude.

Figure 1 shows a schematic diagram of a regular sinusoidal ripple pattern with wave vector aligned parallel to the projection of the incident ion flux of density *J*. Ion flux is incident in the *xOz* plane at an angle *θ* with respect to normal of the mean surface plane (the *Oz* axis) at any arbitrary point, *O*, on the surface. The gradient of the surface ∂*h*/∂*x* is given by tan $\alpha =\left(\frac{\partial h}{\partial x}\right)$, where *α* is the angle between the local surface normal and the *Oz* direction.

Following Carter, under the assumption of small local surface gradient everywhere, the fractional change in sputter erosion rate (with respect to a plane surface) can be expressed as follows:

(1) $$\begin{array}{l} F=\left[\text{sec}\theta /Y\left(\theta \right)\right]\\ \;\left[2\pi^{2}a\left(\theta \right)\;{\left(h_{0}/\lambda \right)}^{2}+6\pi^{4}b\left(\theta \right)\;{\left(h_{0}/\lambda \right)}^{4}+12\pi^{6}c\left(\theta \right)\;{\left(h_{0}/\lambda \right)}^{6}+\ldots \right], \end{array}$$

where *Y*(*θ*) is the sputtering yield, and the coefficients *a*(*θ*), *b*(*θ*), and *c*(*θ*) are functions of cos*θ*, sin*θ*, and sputtering yield *Y*(*θ*) and its derivatives. Thus, fractional change in sputtering yield becomes a polynomial function of even powers of *h*_0_/*λ*.

As the *h*_0_/*λ* ratio increases with continuous ion bombardment, the local angle of incidence, (*θ-α*), along the ripple patterns will eventually become so large that the upstream part of the ripples will be shadowed from the incoming ion flux by the preceding peak. Thus, the limiting condition to avoid such shadowing of incident beam is [26]:

(2) $$\text{tan}\;\left(\pi /2-\theta \right)\geq 2\pi h_{0}/\lambda .$$

According to this condition, if the ratio (*h*_0_/*λ*) exceeds a threshold value, troughs of a sinusoid will not be eroded further but instead erosion will take place at the crests. This in turn may give rise to a sawtooth-like waveform.

## Methods

**Figure 2:**
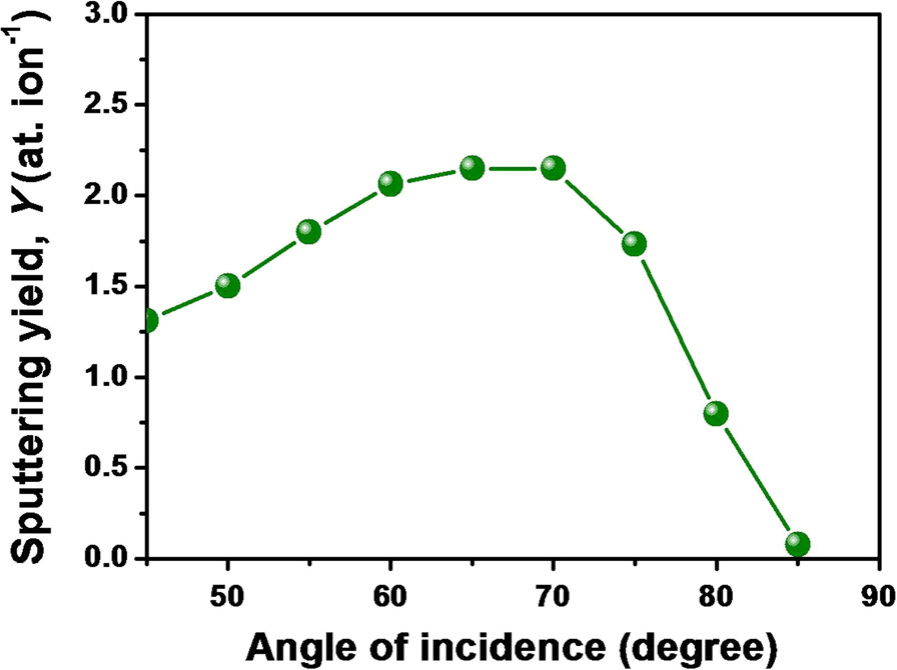
**TRIDYN simulation result.** Showing the variation of sputtering yield of silicon with ion incidence angle (for 500 eV argon ions).

The substrates used in the experiments were cut from a Si(100) wafer. A UHV-compatible experimental chamber (PREVAC, Rogów, Poland) was used which is equipped with a five-axes sample manipulator and an electron cyclotron resonance-based broad beam, filamentless ion source (Tectra GmbH, Frankfurt, Germany). The chamber base pressure was below 5 × 10^-9^ mbar, and the working pressure was maintained at 2.5 × 10^-4^ mbar using a differential pumping unit. Silicon samples were fixed on a sample holder which was covered by a sacrificial silicon wafer of the same lot to ensure a low impurity environment. The beam diameter and the fixed ion flux (throughout this study) were measured to be 3 cm and 1.3 × 10^14^ ions cm^-2^ s^-1^, respectively. Corresponding to this flux value of 500 eV argon ions, the rise in sample temperature is nominal, and hence for all practical purposes, sample temperature should not be very high from room temperature. Experiments were performed for fluences in the range of 1 to 20 × 10^17^ ions cm^-2^ and at two different ion incidence angles, namely 70° and 72.5° (with respect to the surface normal). According to the TRIDYN simulation [33] (as shown in Figure 2), although the sputtering yield maxima is close to 70°, for the sake of completion, we also performed measurements at 72.5° which is not far off from the sputtering yield maxima, and at this higher angle, the shadowing effect is expected to be more prominent.

Following Ar ion exposure, the samples were imaged by *ex situ* atomic force microscopy (AFM). Silicon probes were used having a diameter of approximately 10 nm. Root mean square (rms) surface roughness, *w*, and two-dimensional (2D) autocorrelation function were calculated for all AFM images using the WSxM software [34]. Wavelength of ripple patterns was calculated from the respective autocorrelation functions. As far as faceted structures are concerned, instead of wavelength, we considered the average base width value which was calculated from a large number of line profiles drawn on the respective AFM images. In addition, Rutherford backscattering spectrometric and X-ray photoelectron spectroscopic measurements were performed on Ar ion-bombarded Si samples which did not show the presence of any impurity above their respective detection limits.

## Results and discussion

**Figure 3:**
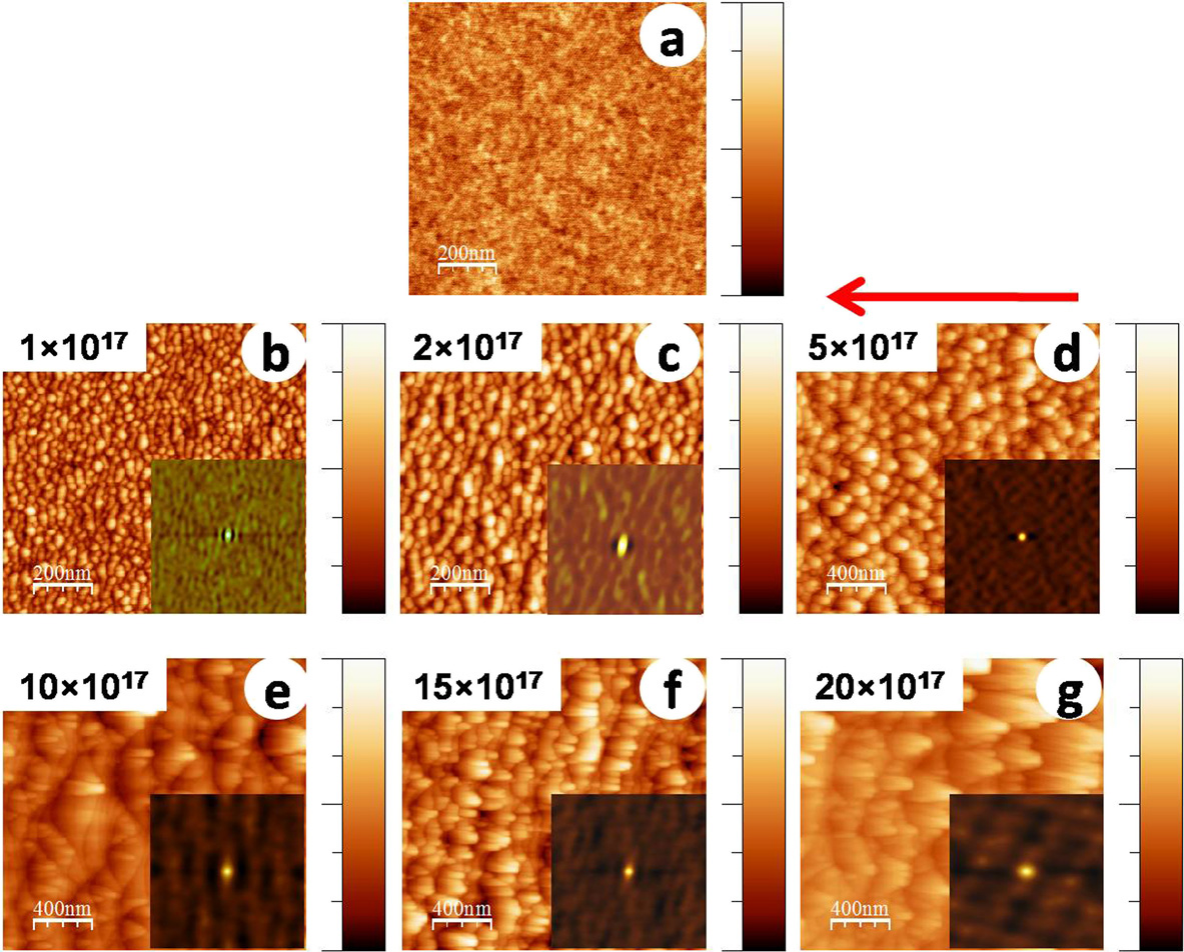
**AFM topographic images obtained from silicon samples.** (**a**) Pristine silicon and those exposed to 500 eV argon ions at an incidence angle of 70° to various fluences: (**b**) 1 × 10^17^, (**c**) 2 × 10^17^, (**d**) 5 × 10^17^, (**e**) 10 × 10^17^, (**f**) 15 × 10^17^, and (**g**) 20 × 10^17^ ions cm^-2^, respectively. The corresponding height scales for (a to g) are the following: 1, 4.3, 9.9, 39.5, 85.7, 60.9, and 182.2 nm. For clarity, (a to c) represent images acquired over a scan area of 1 × 1 μm^2^, whereas (d to g) are of scan area 2 × 2 μm^2^. Insets show the 2D autocorrelation functions for corresponding images.

Figure 3a,b,c,d,e,f,g presents AFM topographic images obtained from silicon samples before and after exposure to argon ion incidence angle 70° at different fluences. Figure 3a presents the AFM image of the pristine sample which shows a smooth surface (rms surface roughness = 0.09 nm). Figure 3b,c shows the signature of corrugated surfaces formed at low fluences, namely 1 × 10^17^ and 2 × 10^17^ ions cm^-2^, respectively. However, small mound-like entities also start appearing on the corrugated surface at the latter fluence. Figure 3d,e,f,g depicts AFM images where mound formation becomes predominant (at the fluence of 5 × 10^17^ ions cm^-2^) which transforms into faceted structures corresponding to the fluence of 10 × 10^17^ ions cm^-2^ and grows further at even higher fluences.

**Figure 4:**
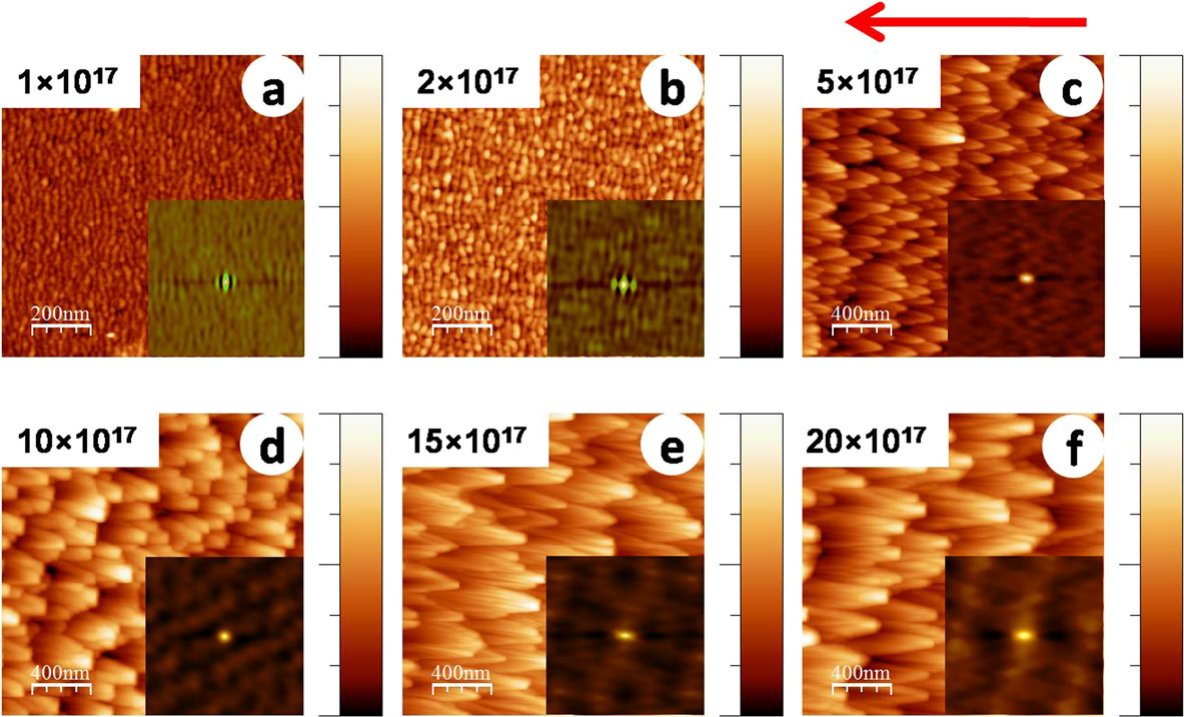
**AFM images of silicon exposed to 500 eV argon ions at 72.5° incidence angle.** At fluences of (**a**) 1 × 10^17^, (**b**) 2 × 10^17^, (**c**) 5 × 10^17^, (**d**) 10 × 10^17^, (**e**) 15 × 10^17^, and (**f**) 20 × 10^17^ ions cm^-2^, respectively. The corresponding height scales for (a to f) are the following: 4, 3.6, 73.9, 85.9, 165.2, and 154.1 nm. For clarity, (a, b) have a scan size of 1 × 1 μm^2^, whereas (c to f) have a scan size of 2 × 2 μm^2^. Insets show the 2D autocorrelation functions for corresponding images.

Figure 4a,b,c,d,e,f shows AFM topographic images corresponding to incidence angle of 72.5° where the presence of ripple morphology is clear up to a fluence of 1 × 10^17^ ions cm^-2^. Beyond this fluence, ripples disappear and small mounds as well as faceted structures evolve (which grow further with increasing fluence) which is evident from Figures 4b,c,d,e,f.

The insets of all the images shown in Figures 3 and 4 represent corresponding 2D autocorrelation functions. In Figure 3, ripple anisotropy is clearly observed at the fluence of 1 × 10^17^ ions cm^-2^, whereas the same in Figure 4 is evident up to the fluence of 2 × 10^17^ ions cm^-2^. The average values (calculated from the AFM images shown in Figures 3 and 4) of ripple wavelength, feature height, and base width of mounds/facets are listed in Table 1 for different fluence values. An increasing trend in height and base width of mounds/facets is observed for both angles of incidence with increasing Ar ion fluence albeit the effect is more prominent at 72.5°.

**Table 1:** **Calculated values of ripple wavelength ( *λ *), feature height ( *h *), and base width of mounds/facets**

**Angle of incidence**	**Fluence (ions cm**^**-2**^**)**	***λ *(nm)**	**Average feature height (nm)**	**Average base width (nm)**
70°	1 × 10^17^	34	2	-
	2 × 10^17^	57	5	-
	5 × 10^17^	-	16	131
	10 × 10^17^	-	22	152
	15 × 10^17^	-	30	199
	20 × 10^17^	-	56	357
72.5°	1 × 10^17^	26	1	-
	2 × 10^17^	27	2	-
	5 × 10^17^	-	28	237
	10 × 10^17^	-	50	363
	15 × 10^17^	-	78	486
	20 × 10^17^	-	90	525

To explain the transition from a rippled surface to faceted structures, we invoke the shadowing condition stated in Equation 2. Let us first consider the case of 70° and the fluence of 1 × 10^17^ ions cm^-2^ where the calculated value of 2*πh*_0_/*λ* turns out to be 0.369, whereas tan(*π*/2 - *θ*) is 0.364. Thus, 2*πh*_0_/*λ* is slightly above the limiting condition which indicates the shadowing effect to start playing a role at this fluence itself. In the case of 2 × 10^17^ ions cm^-2^, the shadowing effect becomes more prominent since 2*πh*_0_/*λ* turns out to be 0.551. As a result, crests of the ripples should undergo more erosion compared to troughs, and hence, there is a likelihood of mounds/facets to evolve. This explains the observation of mounds at this fluence. Similar behaviour is observed in the case of 72.5°. For instance, in the case of 1 × 10^17^ ions cm^-2^, 2*πh*_0_/*λ* equals to 0.242, while tan(*π*/2 - *θ*) turns out to be 0.315. Thus, the condition for no shadowing, i.e. tan(*π*/2 - *θ*) ≥ 2*πh*_0_/*λ* gets satisfied here, and ripples are expected to be seen. The observation of sinusoidal ripples in Figure 4a supports this theoretical prediction. On the other hand, shadowing sets in at the fluence of 2 × 10^17^ ions cm^-2^ since in this case tan(*π*/2 - *θ*) becomes smaller than 2*πh*_0_/*λ* (=0.465). This leads to the formation of small mound-like entities (in the form of broken ripples) appearing on the corrugated surface.

**Figure 5:**
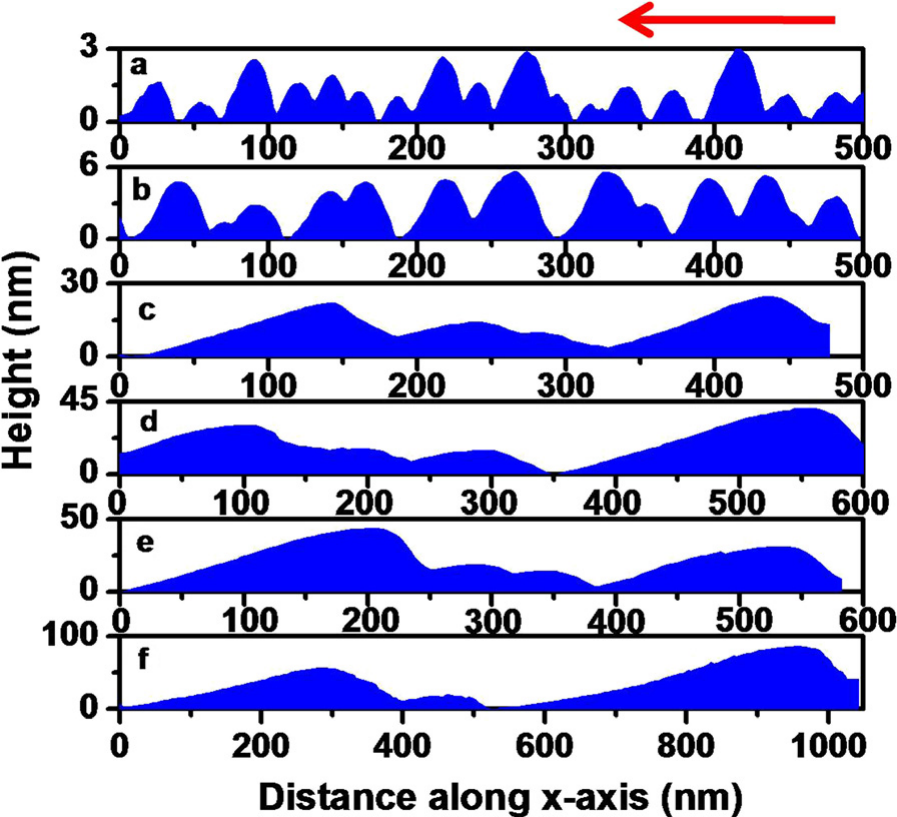
**Line profiles extracted from the AFM images of ion-exposed samples at 70°.** Various fluences: (**a**) 1 × 10^17^, (**b**) 2 × 10^17^, (**c**) 5 × 10^17^, (**d**) 10 × 10^17^, (**e**) 15 × 10^17^, and (**f**) 20 × 10^17^ ions cm^-2^, respectively. Arrow indicates the direction of ion beam onto the surface.

**Figure 6:**
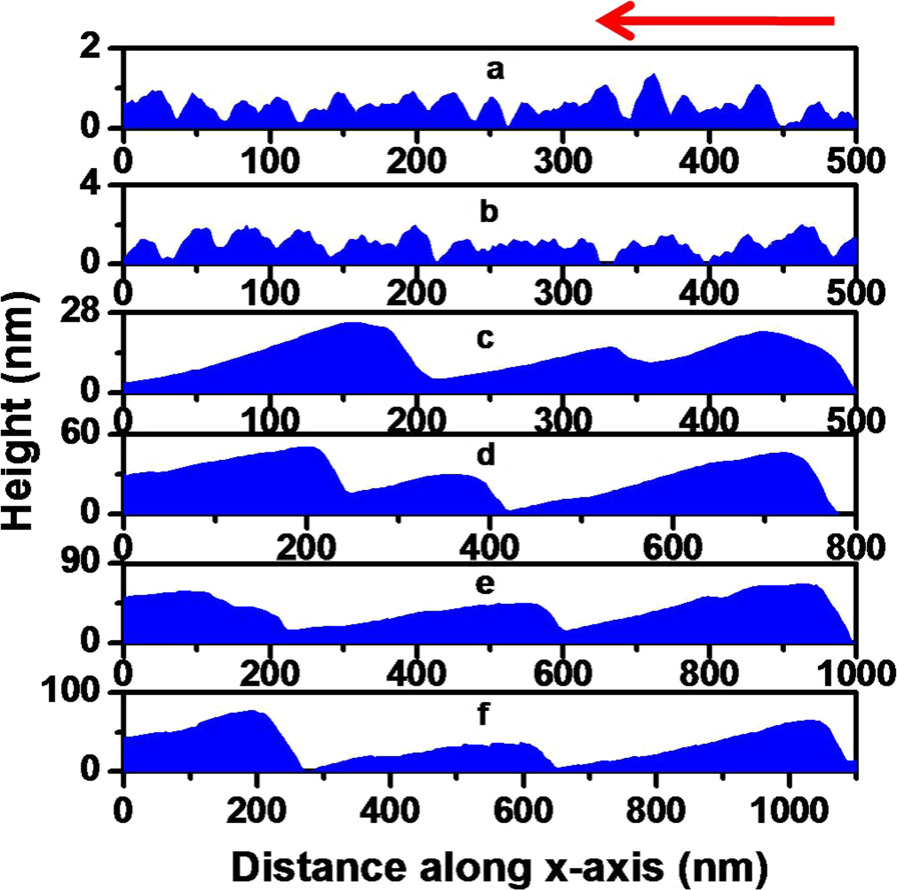
**Line profiles extracted from the AFM images of ion-exposed samples at 72.5°.** Different ion fluences: (**a**) 1 × 10^17^, (**b**) 2 × 10^17^, (**c**) 5 × 10^17^, (**d**) 10 × 10^17^, (**e**) 15 × 10^17^, and (**f**) 20 × 10^17^ ions cm^-2^, respectively. Arrow indicates the direction of ion beam onto the surface.

For further investigation on the role of shadowing effect in morphological evolution, we extracted line profiles of the observed structures along the direction of incident ion beam onto the surface as shown by the arrow marks on the respective AFM images. Line profiles obtained from Figures 3b,c and 4a,b are shown in Figures 5 and 6, respectively. It is observed from Figures 5b and 6b that at the beginning of shadowing transition, the line profiles are still sinusoidal in nature. As discussed previously, beyond shadowing transition, one would expect signature of sawtooth-like waveform. The fact that for both incidence angles sawtooth-like waveform is not yet formed may be attributed to early stage of shadowing where *h*_0_/*λ* ratios are very close to the limiting values or little above. To check this, line profiles obtained from Figures 3d and 4c (corresponding to a higher fluence of 5 × 10^17^ ions cm^-2^) are shown in Figures 5c and 6c which clearly show a transition to sawtooth-like waveform. This is due to the fact that *h*_0_/*λ* ratios (in both cases) are well beyond the respective shadowing limits (0.767 and 0.741, respectively). Thus, we can infer that the effect of ion beam shadowing plays a dominant role in the transition from rippled surfaces to faceted structures and is expectedly more prominent for the higher incidence angle as is evident from the previous discussion.

We now go on to explain the coarsening behaviour of faceted structures (as is evident from Table 1) at higher fluences (>5 × 10^17^ ions cm^-2^) using the mechanism proposed by Hauffe [32]. In this framework, the intensity of reflected ions impinging on an arbitrary area on a facet depends on the dimensions of the reflecting adjoining facets. According to *V*_*n*_ ~ *jY*, where *j* is the ion density on the surface element (which also contains the reflected ions), *Y* is the sputtering yield, and *V*_*n*_ is the displacement velocity of a surface element in the direction of its normal, it is clear that the displacement velocity will be higher for the larger facet. This does not require a particular form of spatial distribution of reflected ions albeit it is necessary that the reflected ions should fall on the neighbouring facets. Accordingly, a smaller facet will disappear into the next bigger one and form an even bigger facet. This corroborates well with the cross-sectional line profiles corresponding to faceted structures shown in Figures 5d,e,f and 6d,e,f which reveal clear enhancements in lateral dimension and height of the faceted structures with increasing ion fluence.

**Figure 7:**
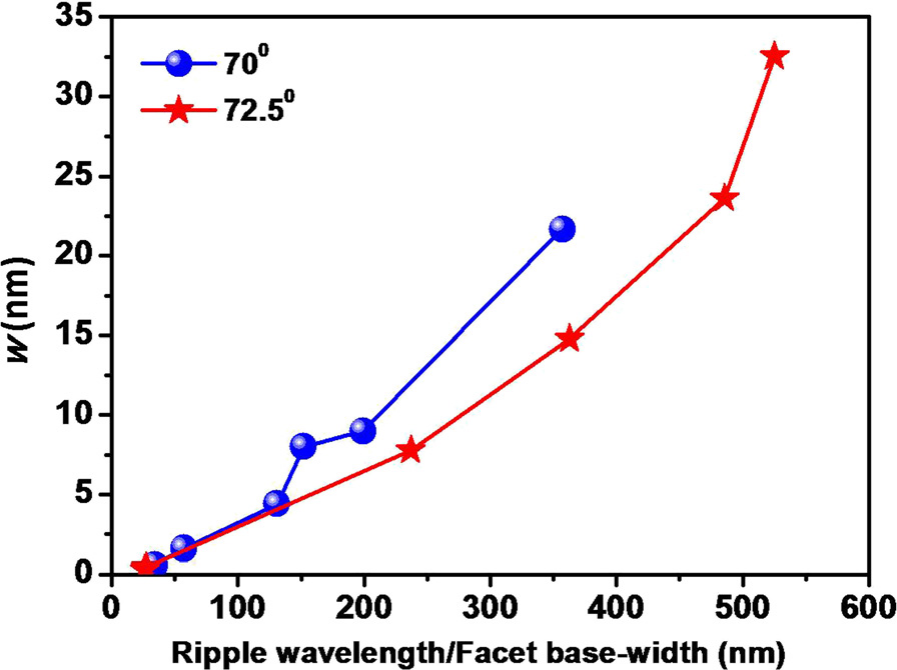
**Variation in rms surface roughness (*w*) with lateral feature dimension corresponding to both angles of incidence.**

The formation of faceted structures and their coarsening behaviour discussed previously are beyond the scope of linear stability analysis of B-H theory because of the presence of ion beam shadowing and possible slope-dependent non-linear effect. In the linear regime, based on Sigmund’s theory of sputtering [35], B-H theory takes into account a competition between curvature-dependent sputtering and surface diffusion. Sputtering is treated as a surface roughening mechanism in this theory, and hence, it is always useful to study the temporal evolution of surface roughness under ion beam erosion to address pattern formation. Figure 7 presents the roughness spectrum (i.e. variation in surface roughness with ripple wavelength/facet base width) for both the angles under consideration. In both cases, we observe an increase in roughness with increasing feature (ripple/facet) dimension.

**Figure 8:**
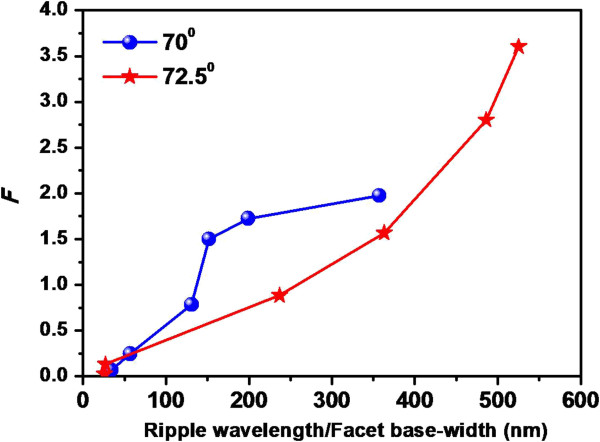
**Variation in fractional change in sputtering yield (*F*).** With lateral feature dimension corresponding to both angles of incidence.

It may be noted that in our case, the sputtering yield would not remain the same due to the evolution of structures having high aspect ratio. According to Carter, the shadowing transition is independent of *Y*(*θ*) and is purely geometric in nature albeit the role of sputtering may not be ruled out. The fractional change in sputtering yield with respect to the flat surface (to begin with) is described in ‘Theoretical approach’. Under this framework, we examine the role of sputtering using Equation 1 which is solved by assuming the dependence: *Y*(*θ*) = *Y*(0) sec*θ*[35]. Although this form is known to be reasonable for not too large values of *θ*, in our case, this approximation simplifies the sputtering yield calculation and explains our results qualitatively. The variation in fractional change in sputtering yield, *F*, with ripple wavelength/facet base width is shown in Figure 8 for both the angles under consideration. It is observed from Figure 8 that *F* follows nearly the similar trend as observed in the case of surface roughness (although a slight mismatch is observed in the case of 70°). Therefore, results shown in Figures 7 and 8 can be considered to be well correlated and confirm our claim that evolution of faceted structures at higher angles of incidence may also be driven by significant contribution from the sputter erosion-induced roughening phenomenon.

## Conclusions

In summary, temporal evolution of surface topography has been systematically studied for silicon under 500 eV Ar ion bombardment for two angles of incidence, namely 70° and 72.5°. For both angles of incidence, parallel-mode ripples are formed at lower fluences which subsequently undergo a transition from parallel-mode ripples to mound/faceted structures. This transition from ripples to mounds and/or faceted structures is explained geometrically which takes into account the inter-peak shadowing effect. Thus, it can be concluded that Carter’s model (mostly used to explain experimental data at intermediate ion energies), applied for the first time in the low ion energy regime, successfully explains the pattern transition observed in the present case. With increasing ion fluence, faceted structures undergo coarsening, i.e. they grow bigger in both lateral dimension and height. The coarsening behaviour is explained by invoking Hauffe’s mechanism which is based on reflection of primary ions on facets. In addition, to check the role of sputtering, fractional change in sputtering yield (with respect to the flat surface) was calculated based on Carter’s theory. It is seen that both fractional change in sputtering yield and surface roughness increase almost in a similar way with fluence-dependent increase in lateral dimension of ripples/facets. Looking into this similar behaviour, it may be concluded that the role of sputtering-induced roughening process cannot be ignored for evolution of ion-induced self-organized patterns.

## Competing interests

The authors declare that they have no competing interests.

## Authors’ contributions

TB wrote the paper and performed irradiation experiments, atomic force microscopy, and other analysis. DPD performed some additional experiments followed by critical data analysis and helped during the manuscript preparation. TS and DPD incorporated the final corrections into the manuscript. All authors read and approved the final manuscript.
